# Importin 7 mediates the nuclear import of HIV‐1 integrase via a specific interacting interface

**DOI:** 10.1002/2211-5463.70294

**Published:** 2026-07-06

**Authors:** Juana Bana, Avigail Yariv, Tal Oppenheim, Hadar Amartely, Dana Reichmann, Oded Livnah

**Affiliations:** ^1^ The Wolfson Centre for Applied Structural Biology The Edmond J. Safra Campus, The Hebrew University of Jerusalem Israel; ^2^ Department of Biological Chemistry, Alexander Silverman Institute of Life Sciences, The Edmond J. Safra Campus The Hebrew University of Jerusalem Israel; ^3^ The Center for Nanoscience and Nanotechnology, Safra Campus Givat Ram The Hebrew University of Jerusalem Israel; ^4^ Present address: The Max Planck Institute of Biophysics Frankfurt am Main Germany

**Keywords:** HIV‐1, karyopherins, nuclear localization signal, nucleocytoplasmic transport, protein–protein interactions, viral infection

## Abstract

HIV‐1 integrase (IN) must cross the nuclear envelope to access the host genome and catalyze viral DNA integration, a process that requires active nuclear import. Although recent work has established that the primary pathway for HIV‐1 nuclear entry involves transport of the intact capsid, complementary mechanisms may enable the nuclear import of individual viral components through the host karyopherins. Among the host nuclear import factors implicated in this process, importin 7 (Imp7) has emerged as a strong candidate for IN, yet its precise role and the molecular basis of its interaction with IN have remained unclear. Here, we demonstrate that Imp7 acts as a critical mediator of IN nuclear import. Using hydrogen–deuterium exchange and cross‐linking mass spectrometry, we map the interaction interface and identify the core domain of Imp7 as the primary IN binding site. Affinity measurements reveal high‐affinity binding between IN and Imp7, while mutations within the C‐terminal nuclear localization signal of IN significantly weaken this interaction. Consistent with these findings, knockdown of endogenous Imp7 in HEK293T cells leads to cytoplasmic accumulation of IN, confirming its essential role in nuclear import. Together, these results establish a direct, high‐affinity interaction between IN and Imp7, define their molecular interface, and position Imp7 as a key nuclear import receptor for HIV‐1 IN. This work provides detailed molecular insight into a critical host–virus interaction during early HIV‐1 replication and highlights the Imp7‐IN interface as a promising target for complementary therapeutic intervention aimed at disrupting integrase nuclear import.

## Graphical abstract

HIV‐1 integrase enables viral DNA integration into the host genome. By binding to the core domain of the host protein Importin 7 via its C‐terminal domain, the integrase is transported across the nuclear membrane into the nucleus, where integration of the viral genome into host DNA takes place. This translocation is a critical step for subsequent viral genome integration.

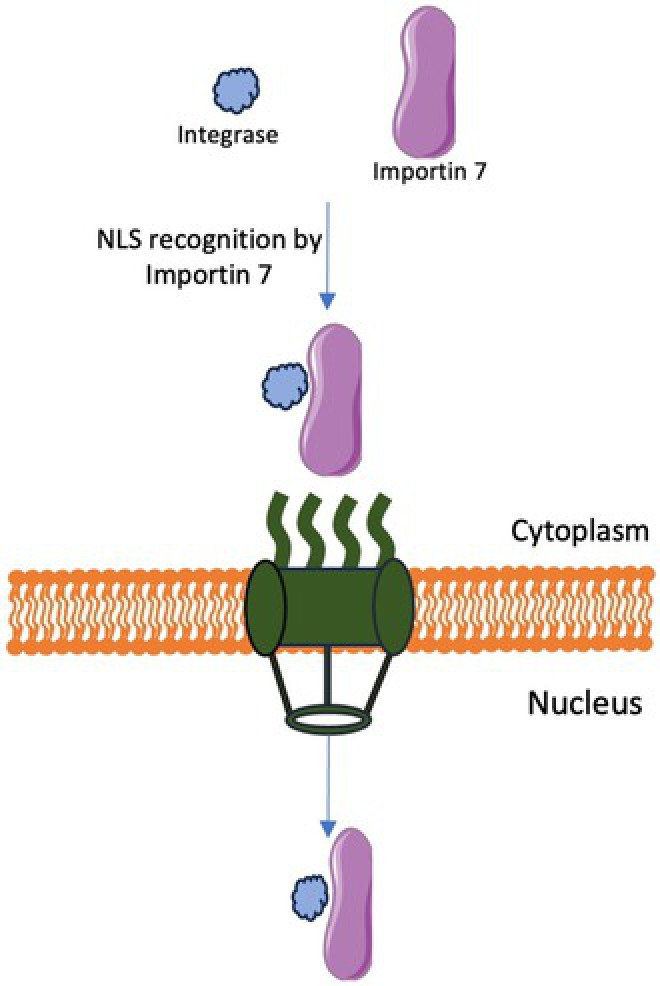

**Abbreviations**
DTT: Dithiothreitol
HDX‐MS: hydrogen deuterium mass spectrometry
HIV‐1: human Immunodeficiency virus type 1
Imp7: importin 7
Impα: importin α
Impβ: Importin β
IN‐CTD: integrase C‐terminal domain
LC–MS/MS: Liquid Chromatography–Tandem Mass Spectrometry
TCEP: Tris(2‐carboxyethyl)phosphine hydrochloride
XL‐MS: crosslinking mass spectrometry

Nucleocytoplasmic transport is the rapid and selective exchange of macromolecules between the nucleus and the cytoplasm in both directions across the nuclear envelope [1, 2, 3] (Fig. 1). This evolutionary conserved mechanism is essential to numerous cellular functions and relies on very‐well coordinated interactions among multiple proteins. For efficient nuclear transport, proteins and mRNAs bind to soluble carrier proteins termed Nuclear Transport Receptors (NTRs). Many of these NTRs belong to the diverse family of karyopherin proteins, mediating transport of cargo molecules into and out of the nucleus through the nuclear pore complex (NPC) [4, 5]. Karyopherins can be divided into two subfamilies comprising of karyopherins α and karyopherins β [6] where both karyopherin subfamilies share similar topologies of helical repeats that form a superhelical tertiary structure (Fig. 2A). However, each of the subfamilies are composed by different types of repeating motifs. Karyopherins α are constructed of armadillo (ARM) repeat motifs, whereas karyopherins β proteins are constructed of HEAT repeats. In this context, each ARM repeat motif consists of three helices, whereas a single HEAT repeat motif consists of two helices, forming a helical hairpin [6, 7, 8]. Among the most extensively studied karyopherins are importin α (Impα) and β (Impβ), which transport a wide range of cargoes across the nuclear membrane [9, 10]. Impα and Impβ function either independently or as a complex [11, 12], engaging with specific cargos through nuclear localization signals (NLSs) or nuclear export signals (NESs) [13, 14, 15, 16, 17]. Another identified karyopherin that mediates nuclear transport as a complex with Impβ is importin 7 (Imp7). Impβ/Imp7 heterodimer was previously shown to facilitate the transport of H1 histone [18, 19]. In addition, Imp7 was found to transport many other protein cargoes into the nucleus such as hTERT, p38, SMAD and others [20, 21, 22, 23, 24]. In addition to their natural cargoes, karyopherins are sometimes being utilized by viral proteins that must enter the host nucleus facilitating viral infection. A notable example of this is the viral proteins of the Human Immunodeficiency Virus Type 1 (HIV‐1).

**Fig. 1:**
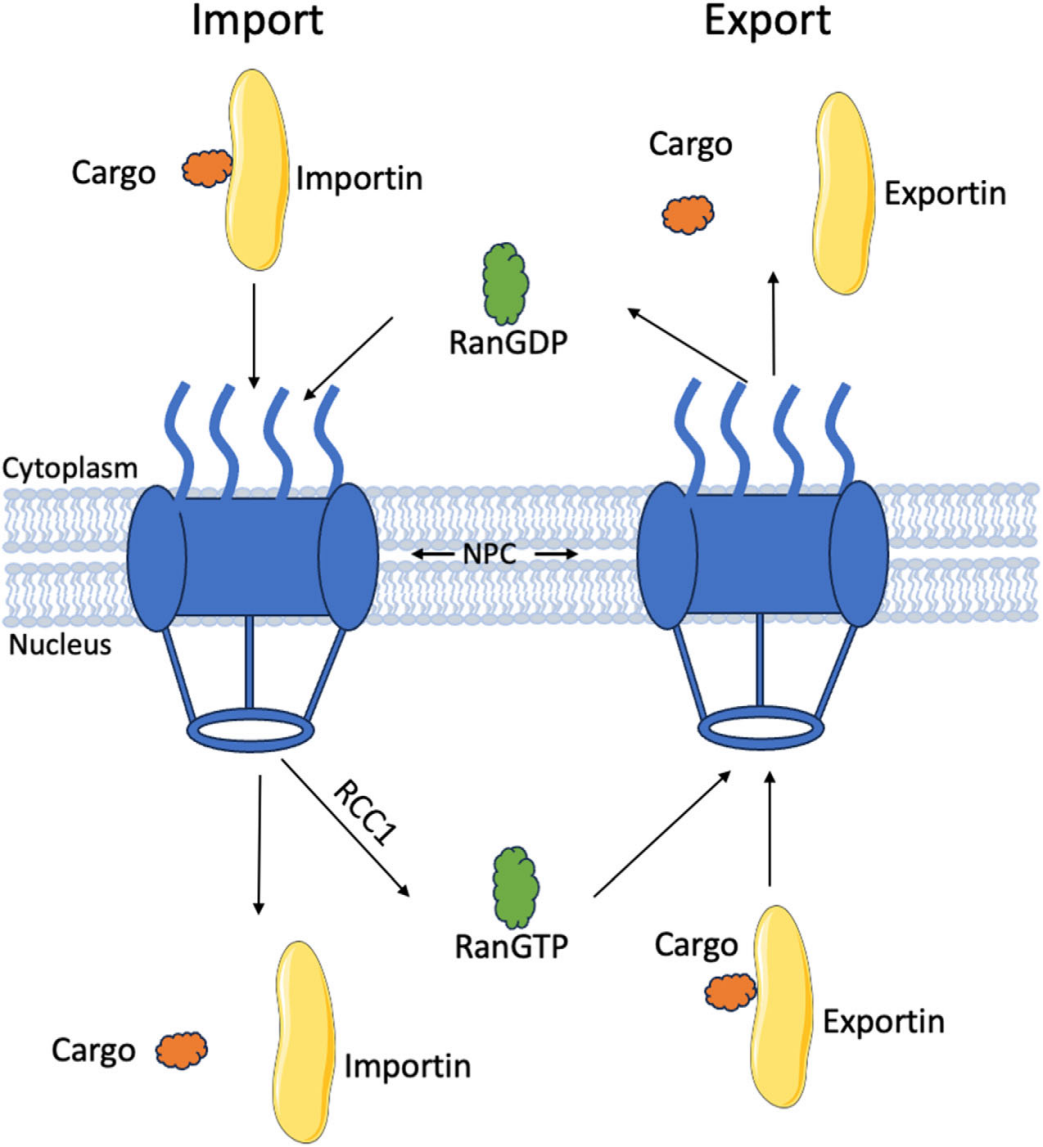
Nuclear import and export. Proteins from the karyopherin family mediate the transport of cargoes between the cytoplasm and the nucleus. During the import process, importins recognize their cargoes via nuclear localization signals (NLS) and bind them. The importin–cargo complex then traverses the nuclear envelope through interactions with the nuclear pore complex (NPC). Once inside the nucleus, the complex binds to RanGTP, causing the release of the cargo and dissociation of the complex. The export process is similar, except that exportins bind to RanGTP in the nucleus before transporting the cargo across the nuclear envelope.

**Fig. 2:**
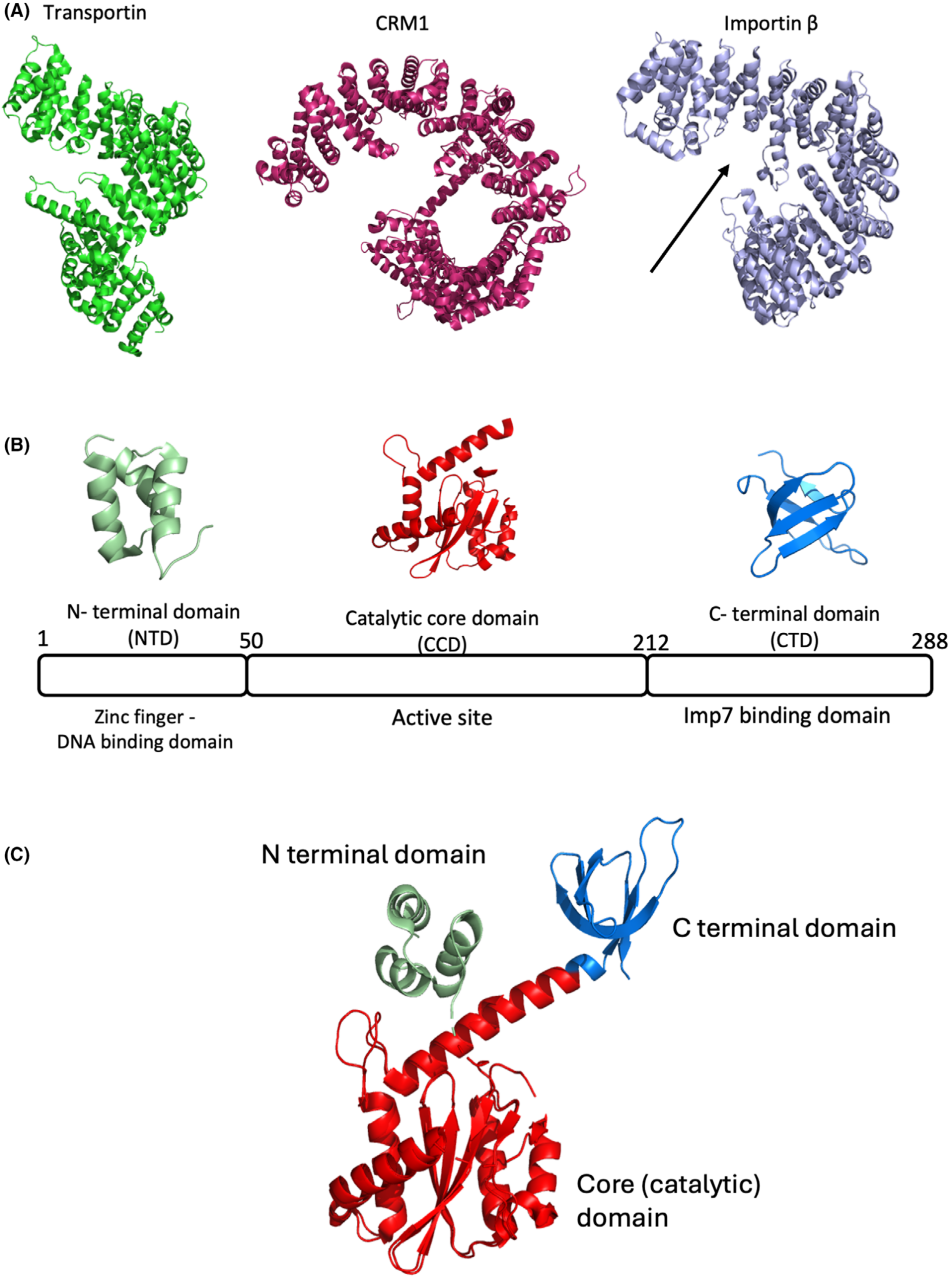
Cartoon representation of the structures of proteins belonging to the karyopherins family and HIV‐1 integrase. (A) Transportin (left, green PDB ID:2QMR), Crm1 (middle, dark pink, PDB ID:4FGV) and importin β (right, light blue, PDB ID:4XRI) belong to the subfamily of karyopherin β. The typical structure of karyopherins β is superhelical arrangement of 18–20 tandem repeats of the HEAT motif forming a central core region within the protein that typically serves as the cargo‐binding site (indicated by a black arrow in importin β). (B) The HIV‐1 integrase comprises three domains: the N‐terminal domain that has a zinc finger motive and responsible for DNA binding; the catalytic domain, accountable for the enzyme's catalytic activity postentry into the host nucleus; and the C‐terminal domain, facilitating protein interactions with Imp7. (C) The three‐dimensional structure of HIV‐1 integrase protein. The complete protein structure was constructed using the data from PDB entries 1K6Y (covering the N‐terminal domain and the core domain) and 1EX4 (covering the core and the C‐terminal domains). The two structures were aligned based on their overlapping core domain. All the structures were visualized using PyMol.

HIV‐1 is known for its rapid and irreversible infection of host cells, and unlike many retroviruses, HIV‐1 is capable of infecting nondividing cells, where the nuclear envelope remains intact [25]. As a result, HIV‐1 utilizes the active nuclear import mechanisms of the host cell to transport its components into the nucleus [26, 27, 28]. To harness the cell's machinery effectively, HIV‐1 relies on its own proteins, which must enter the nucleus of its host. A known example of an HIV‐1 protein that uses the host cell machinery is Rev protein, that has been discovered to interact with Impβ, promoting its entry into the host cell nucleus during the later stages of infection [29, 30]. Initially, it was assumed that the HIV‐1 capsid uncoating, that includes nucleocapsid proteins, reverse transcriptase, and integrase, occurs exclusively in the cytoplasm [31, 32]. However, recent studies suggest that uncoating may also take place in the nucleus, in which case the viral capsid would enter the nucleus as a complete structure rather than as individual components [33]. A recent study has further explored how the intact HIV‐1 capsid itself can traverse the nuclear pore complex [34]. These new insights emphasize the complexity of nuclear entry and highlight that multiple viral and host factors may contribute to this process. Nevertheless, uncoating can still occur in the cytoplasm, and then the individual capsid components would be transported individually using the host cell nuclear transport proteins. A key HIV‐1 capsid component that utilizes the host cell's nuclear compartment is HIV‐1 integrase (HIV‐1 IN), which enables integration of the viral DNA into the host genome. This process requires that IN cross the double membrane of the cell nucleus. IN contains three distinct functional domains, each playing a crucial role in its function. The N‐terminal zinc‐binding domain (residues 1–50), which contributes to integrase multimerization and DNA binding. The second domain is the catalytic core domain (Residues 51–212), that mediates the cleavage and strand transfer reactions necessary for viral DNA integration. The third is the C‐terminal domain (Residues 213–288), which interacts with both viral and host DNA, playing a key role in the target DNA recognition (Fig. 2B,C). Additionally, the C‐terminal domain is crucial for the nucleocytoplasmic transport of IN by interacting with Imp7, thereby facilitating its intracellular trafficking [35].

Studies have shown that Imp7 enables the nuclear entry of certain components of HIV‐1, such as IN and reverse transcriptase [36, 37]. Specifically, IN interacts with Imp7 primarily through its C‐terminal region, where the NLS of IN is located within two regions: one containing three lysine residues (^235^WKGPAKLLWKG) and the second is an arginine/lysine‐rich region (^262^RRKAK). Moreover, mutations in these sites disrupt IN's ability to bind Imp7 [38, 39]. In addition, viruses produced in host cells lacking Imp7 have a reduced infection rate [38]. Despite the importance of these interactions, their structural characterizations remain unknown. Understanding the interaction between nuclear transport receptors and their cargo is crucial for unraveling the mechanisms of nucleocytoplasmic transport in cells. Moreover, exploring the role of the C‐terminal domain of IN in the nuclear transport of the protein could reveal new therapeutic targets for HIV‐1.

Here, we are focusing on the specific interactions between IN and Imp7 from *Xenopus laevis* (shares 92% sequence identity with human Imp7) providing new insights into their molecular interaction. We identified the core domain of Imp7 as the binding site for IN and demonstrate the critical role of the integrase NLS in this interaction, supported by cross‐linking and affinity measurements. Furthermore, immunofluorescence assays in HEK293T cells reveal that Imp7 is essential for the nuclear import of HIV‐1 IN, as its knockdown leads to cytoplasmic accumulation of the protein. Together, these findings highlight the key role of Imp7 in IN nuclear import, mapping the landscape of the interaction and offering a more comprehensive view for targeting this interaction in antiviral strategies.

## Materials and methods

### Sequence alignment of importin 7

**Fig. 3:**
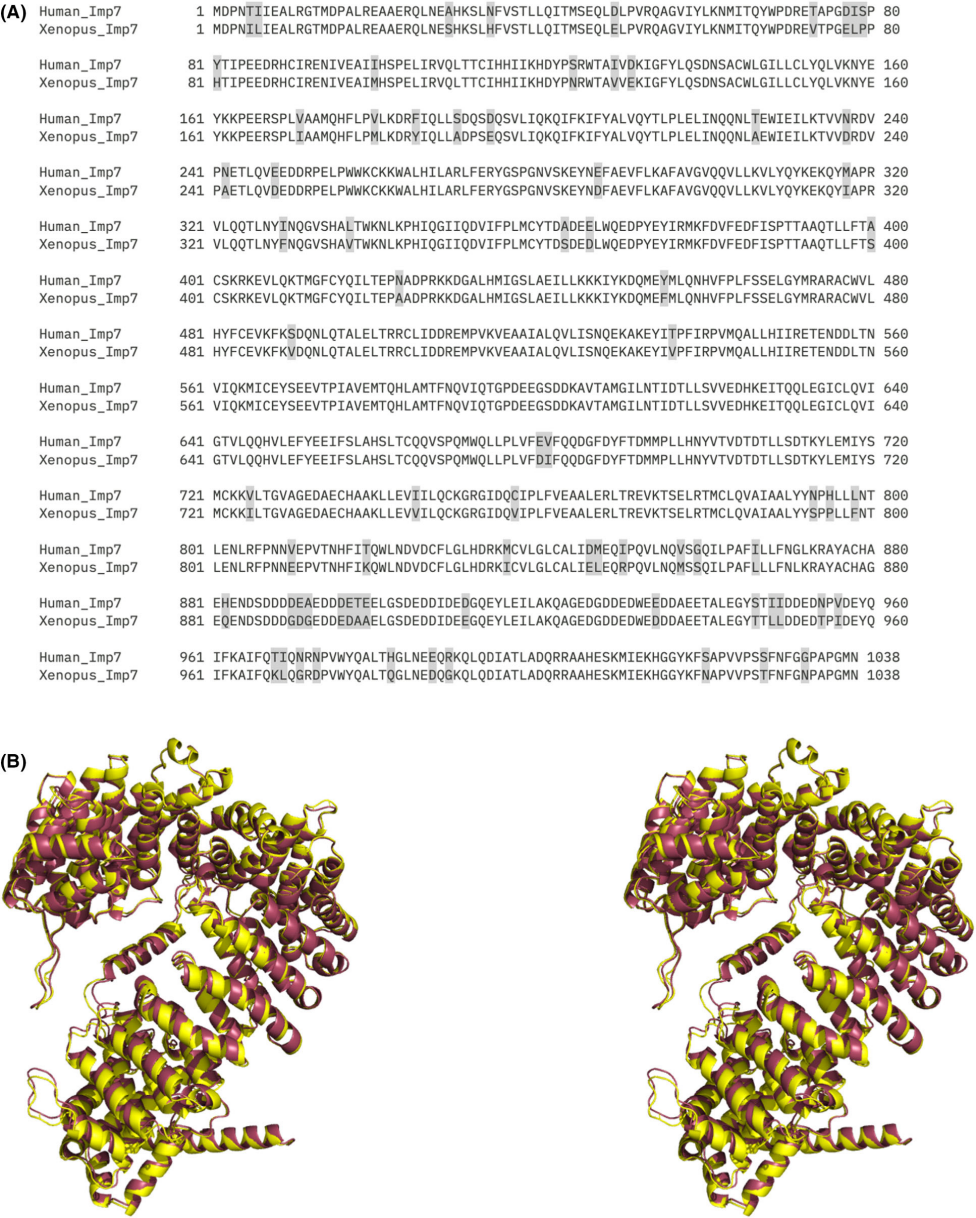
Sequence and structural alignment of human and *Xenopus laevis* Importin 7. (A) Sequence alignment of human and *Xenopus laevis* Imp7 reveals 97% sequence similarity and 92.5% sequence identity. Residues that differ between the two sequences are highlighted in gray, with most substitutions representing conservative changes. (B) Stereo representation of structural alignment of the AlphaFold3 models of human (colored in bordeaux) and *Xenopus laevis* Imp7 (colored in yellow) demonstrates a high degree of structural similarity between the predicted structures, supporting the use of *Xenopus* Imp7 as a model for human Imp7 and suggesting that conclusions drawn from the *Xenopus* protein are most likely applicable to the human homolog. Disordered C‐terminal tails were removed. The sequence alignment was generated using the online tool EMBOSS needle. The structural alignment is visualized using PyMol.

*Xenopus laevis* Imp7 was used for all the described assays (HDX‐MS, XL‐MS, and affinity measurements) in place of the human Imp7. The Xenopus ortholog exhibits better biochemical behavior, including increased stability and higher purification yield, and previous work established its ability to bind IN [38]. Importantly, Xenopus Imp7 shares 92% and 97% sequence identity and similarity, respectively, with human Imp7 (Fig. 3), supporting its suitability as a functional equivalent model in biochemical and structural analyses. The immunofluorescence experiments relied on endogenous human Imp7, as HEK293 cells naturally express this protein. Sequence alignment was performed using EMBOSS needle [40]. The alignment highlights the strong conservation between the two constructs, with sequence differences indicated in gray.

### Protein expression and purification

#### Importin 7

Hexahistid**i**ne‐tagged *Xenopus laevis* Imp7 was cloned into pHis2 plasmid and expressed in *E. coli* C41 strain (DE3; Lucigen, Middleton, WI, USA). After initial growth of the bacteria in 2xYT medium (consisting of 16 g of trypton, 10 g of yeast extract and 5 g of NaCl in 1 L), isopropyl 1‐thio‐β‐D‐galactopyranoside (IPTG) was added for induction and cultivation overnight in 26 °C and 180 rpm. After harvesting, cell resuspension is carried out using buffer that contains 50 mM Tris–HCl pH 8, 300 mM NaCl, 5 mM β‐mercaptoethanol and 10 mM imidazole. Protease inhibitor cocktail (MilliporeSigma, St. Louis, MO, USA) was added to the buffer at a ratio of 1:400 and 2.5 μg lysozyme per 1 L of bacteria. Then, lysis was performed using microfluidizer at 20 K psi (M‐110EHIS; Microfluidics Corporation, Newton, MA, USA) for separation of soluble protein. Purification of the Imp7 consists of two steps. First, initial purification is conducted on Ni^2+^‐NTA column. Elution is pe**r**formed using buffer that contains 50 mM Tris–HCl pH 8.0, 300 mM NaCl, 5 mM β‐mercaptoethanol and 500 mM imidazole. The next step is purification using anion exchange column resource Q15 with buffer A that contains 50 mM Tris–HCl pH 8, 50 mM NaCl, 1 mM DTT, and 5% glycerol and buffer B that contains 50 mM Tris–HCl pH 8, 1 m NaCl, 1 mM DTT, and 5% glycerol. Both steps are conducted on ÄKTA™ explorer (Cytiva, Marlborough, MA, USA). The relevant fractions are then concentrated to 5–6 mg/mL using a 15 mL centricon device (30 000 MWCO, Biological Industries, Beit HaEmek, Israel) and stored at −80 °C.

#### C‐terminal domain of HIV‐1 integrase

The C‐terminal domain of the IN (Residues 220 to 280) will be indicated henceforth as IN CTD, was cloned into pHis2 plasmid and expressed in *E. coli* Rosetta strain (Lucigen, Middleton, WI, USA), in 2xYT that contains 5% glucose and 50 mL of NPS buffer. After initial growth of the protein in the bacteria, IPTG was added for induction and cultivation overnight at 22 °C and 180 rpm. After harvesting, cell resuspension is carried out using buffer that contains 20 mM HEPES pH 7.5, 1 m NaCl, 2 mM β‐mercaptoethanol, 10% glycerol, and 10 mM imidazole. Then, lysis was performed using a microfluidizer at 20 K psi (M‐110EHIS; Microfluidics Corporation, Newton, MA, USA) for separation of soluble protein. Purification of the IN CTD consists of two steps. Initial purification was conducted via a Ni^2+^‐NTA affinity column. Elution performed using buffer that contains 20 mM Hepes pH 7.5, 1 m NaCl, 2 mM β‐mercaptoethanol, 10% glycerol, and 500 mM imidazole. 1 mM EDTA was added to the elution's samples to prevent aggregation of the protein. The second purification step utilized a size exclusion sephacryl S‐100, 500 mL column with buffer that contains 20 mM HEPES pH 7.5, 1 m NaCl, 1 mM DTT, and 5% glycerol. Both steps are conducted on an ÄKTA™ explorer system (Cytiva, Marlborough, MA, USA). The relevant fractions are then concentrated using a 2 mL centricon device (3000 MWCO, Biological Industries, Beit HaEmek, Israel) to 1–2 mg/mL and stored at −80 °C. To explore the impact of the NLS on the affinity, we have generated an additional IN CTD construct, named IN CTD mutant, that has two mutations in the second NLS (changing RRKAK to RAAAK). This construct was expressed and purified using the same procedures as previously described.

#### Purification of the Imp7—IN CTD complex

For the HDX‐MS and XL‐MS we formed a purified sample of the complex. Imp7 and the IN CTD (the latter in excess ratio of 1–1.5) were both incubated together overnight at 4 °C to form a 1:1 complex. Consequently, the complex was dialyzed in buffer containing 40 mM NaCl, 20 mM HEPES pH 7.5, and 1 mM DTT prior to loading to the column. After dialysis, the complex was loaded onto a 3 mL mono Q anion exchange column. The complex eluted in one peak and was confirmed via SDS/PAGE and was subsequently concentrated using 2 mL centricon device with 10 000 MWCO (Biological Industries, Beit HaEmek, Israel) to the final concentration of 2–3 mg/mL and stored at −80 °C.

### HDX‐MS analysis

HDX‐MS experiments were conducted using the automated HDX robot (LEAP Technologies, Fort Lauderdale, FL, USA) coupled to an M‐Class Acquity LC and HDX manager (Waters Ltd., Wilmslow, Manchester, UK). Before the analysis, equilibration buffer (20 mM HEPES pH 7.5, 500 mM NaCl, 1 mM TCEP) was used to dilute sample to 10 μm. 5 μL sample was added to 95 μL deuterated buffer (20 mM HEPES pD 7.5, 500 mM NaCl, 1 mM TCEP) and incubated for 0, 0.5, 2, 10 or 30 min, at 4 °C, with each condition being measured in triplicate (*t* = 0 was acquired with protonated buffer to provide the baseline mass). In order to quench the samples after the labelling reaction, 75 μL of quenching buffer (50 mM potassium phosphate, 0.05% DDM, pH 2.5) was added to 75 μL of the labeled solution. A 50 μL of the quenched sample was loaded onto a home‐packed pepsin column with agarose‐immobilized pepsin (Thermo Fisher Scientific) and processed at a flow rate of 40 μL**·**min^−1^ (20 °C). Digestion proceeded for 3 min on a VanGuard Pre‐column Acquity UPLC BEH C18 (1.7 μm, 2.1 mm × 5 mm, Waters Ltd., Wilmslow, Manchester, UK) using 0.3% formic acid in water. Then, the resulting peptic peptides were loaded to a C18 column (75 μm × 150 mm, Waters Ltd., Wilmslow, Manchester, UK) at 0 °C, and subsequently separated using a gradient elution of 0–40% MeCN (0.1% v/v formic acid) in H_2_O (0.3% v/v formic acid) over 12 min at 40 μL·min^−1^, also at 0 °C. The HDX system was coupled to a Synapt G2Si mass spectrometer (Waters Ltd., Wilmslow, Manchester, UK) operated with electrospray ionization using a capillary voltage of 3 kV, cone gas of 50 L/h, a desolvation gas of 300 L/h, and nebulizer pressure of 6.5 bar. Desolvation and source temperatures were selected to 120° and 80°C, respectively. HDMSE, along with dynamic range extension and sensitivity modes (Data‐Independent Analysis coupled with IMS separation and single reflection time‐of‐flight) were used to separate peptides prior to CID fragmentation. The trap and transfer cells were maintained at an argon pressure of 2.4 × 10^−2^ mbar, while helium and nitrogen pressures were 4.21 mbar and 3 mbar (drift tube), respectively. For the trap, wave heights and velocities were 311 m/s and 4 V. for the transfer cell, the wave heights and velocities were 650 m/s and 30 V for the IMS and 175 m/s and 4 V. Signals were collected over an m/z range of 50–2000 for 0.6 s, alternating between high‐ and low‐energy modes. Peptide fragmentation in the high‐energy scans was achieved using collision energy ramp of 18–40 V. HDX data were analyzed by DynamX (v3.0.0) and PLGS (v3.0.2) available with the mass spectrometer. The identification of peptide was performed using a minimal database comprising the target protein sequences and pepsin. In DynamX, filters were applied as follows: minimum intensity: 10000, minimum products per MS/MS spectrum: 3, minimum products per amino acid: 0.3, maximum sequence length: 18, maximum ppm error: 10, file threshold: 4/6. Manual curation of the datasets were subsequently used to generate woods differential plots in Deuteros 2.0 [41], employing hybrid statistics at a significance level of 0.01.

### Crosslinking of the complex and Nano‐LC–MS/MS analysis

The Crosslinker used in this study was the amine‐amine crosslinker BS3 (bis(sulfosuccinimidyl)suberate), conjugating two amine groups together (e.g., lysines or N‐terminal amines), or amine group with serine (S), tyrosine (Y), or threonine (T). In our case, 40 μm of Imp7‐IN CTD complex was incubated with BS3 (3 mM final concentration) at 25 °C at 350 rpm for one hour. Samples were quenched with Tris–HCl (20 mM final concentration) and then denatured with 6 m urea and 3 mM DTT, for 30 min incubation at 37 °C at 350 rpm. Reduced thiols were alkylated with 20 mM iodoacetamide (IAM) prepared in 6 m urea and incubated for 60 min at 25 °C in the dark with shaking at 350 rpm. The proteins were then digested by trypsin overnight and the reaction was quenched with 0.1% formic acid (FA). Then, samples were loaded onto in‐house made 3‐layer prewashed C18 StageTips [42]. Samples were then washed and eluted. Then, eluted peptides were dried using SpeedVac for 40 min at 1300 rpm at 35 °C and then resuspended with 12 μL of 0.1% HPLC‐grade trifluoroacetic acid (TFA).

Samples were loaded onto Nano Trap Column, 100 μm i.d. × 2 cm, packed with Acclaim PepMap100 C18, 5 μm, 100 Å (Thermo Scientific), and subsequently separated on a C18 reverse‐phase column coupled to the Nano electrospray, EASY‐spray (PepMap, 75 mm × 25 cm, Thermo Scientific) using a Dionex Nano‐HPLC system (Thermo Scientific) coupled online to Orbitrap Mass spectrometer, Q Exactive HP (Thermo Scientific). For peptide separation, the column was operated at 45 °C using a linear gradient, divided into sections of varying length. The Q Exactive HP was operated in a data‐dependent mode. Up to the 10 most abundant isotope patterns with a charge of ≥ 3 and < 8 were subjected to higher‐energy collisional dissociation with a normalized collision energy of 25 and 27, an isolation window of 1.6 m/z, and a resolution of 45 000 at m/z. Dynamic exclusion of sequenced peptides was set to 20 s for limiting repeated sequencing. Ion injection time and ion target value thresholds were set to 20 milliseconds (ms) and 3 × 10^6^ for the survey scans and to 30 ms and 10^5^ for the MS/MS scans. Only ions with ‘peptide preferable’ profile were analyzed for MS/MS. Xcalibur software (Thermo Scientific) acquired all data.

The proteomics data for both mass spectrometry experiments have been deposited to the ProteomeXchange Consortium (http://proteomecentral.proteomexchange.org) via the PRIDE partner repository [43], with the dataset identifier PXD066885 for the XL‐MS data and PXD068263 for HDX‐MS data.

### Affinity measurements

The K_d_ values of the interactions of the Imp7 and IN CTD (wt and mutant) complexes were determined by the biolayer interferometry method using a GatorPrime (GatorBio, Königswinter, Germany). For each IN CTD construct, the assay was performed in black 96‐well plates. Biosensors coated with carboxyl groups for attaching amine groups were activated with a mixture of 20 mM EDC (1‐ethyl‐3‐(3‐dimethylaminopropyl) carbodiimide hydrochloride) and 10 mm N‐hydroxysuccinimide (NHS) followed by conjugation of 5 μm of IN CTD construct in acetate buffer pH 5. Then, 200 μL of ethanolamine was used for quenching. After quenching, association of IN CTD construct with Imp7 in a concentration range of 1–40 000 nm was performed. K_d_ values were determined by steady‐state (equilibrium) analysis, in which the equilibrium response signals at multiple protein concentrations were measured and fitted to a Michaelis–Menten binding model. The binding data was collected at 25 °C. For each IN CTD construct, two technical replicates were performed.

### Cell culture, transfection, knockdown, and immunofluorescence assay

HEK293T cells were cultured in DMEM supplemented with 10% fetal bovine serum (FBS), 100 U/mL penicillin, 100 μg/mL streptomycin, and 1% glutamate at 37 °C in a humidified atmosphere containing 5% CO_2_. Before transfection/knockdown, cells were seeded onto glass coverslips in a 6‐well plate at a density of ~ 50 000 cells/well and allowed to adhere overnight. Cells were transfected with pTwist EF1 Alpha Puro for expressing integrase (the full length of integrase was used for the immunofluorescence experiments) (Twist bioscience) or cotransfected in addition to the plasmid with siRNA targeting the Imp7 gene using transitX2 according to the manufacturer's protocol (Mirus). For plasmid transfection, 5 μg of DNA was mixed with 7.5 μL of the transfection reagent in serum‐free medium and incubated for 30 min at room temperature before adding it to the cells. Cells were incubated for 48 h post‐transfection. For knockdown of Imp7 and transfection of integrase experiments, 25 nm of siRNA and 5 μg of DNA were used, and cells were incubated for 72 h post‐transfection to allow gene silencing.

Following incubation time, the cells were then fixed with 4% paraformaldehyde for 30 min at room temperature, followed by permeabilization and blocking with 0.1% Triton X‐100, 1% BSA, and 10% FBS in PBS for 1 h. Then, the cells were incubated for 3 h at room temperature with primary antibodies against Imp7 and IN (ab115208, ab66645, Abcam), diluted 1:250 in the blocking solution. Subsequently, the cells were washed three times with PBS and incubated with secondary antibodies (1:700 dilution) for 1 h at room temperature in the dark. Nuclei were counterstained with DAPI (1 μg/mL) for 10 min. The coverslips were mounted onto glass slides using mounting medium, and fluorescence images were captured using the FV‐1200 confocal microscope with a 60X/1.42 oil objective (Olympus, Japan). Multiple dyes sequential scanning mode was used in order to avoid emission bleed‐through. The experiment was performed with two biological replicates that had two technical replicates for each condition. Image analysis was performed using imageJ (National Institutes of Health [NIH]). Nuclear and cytoplasmic fluorescence intensities were quantified, and the nuclear‐to‐cytoplasmic fluorescence ratio was calculated.

The cell line that was used in this study is HEK293/T17 (RRID: CVCL_1926) and was purchased from ATCC (Manassas, VA, USA). The cell line identity was authenticated by the supplier prior to distribution using standard quality‐control procedures. Cells were cultured under the conditions described above. Cells were routinely checked for mycoplasma contamination and all experiments were performed using mycoplasma‐free cells.

## Results

### AlphaFold3 structure predictions

**Fig. 4:**
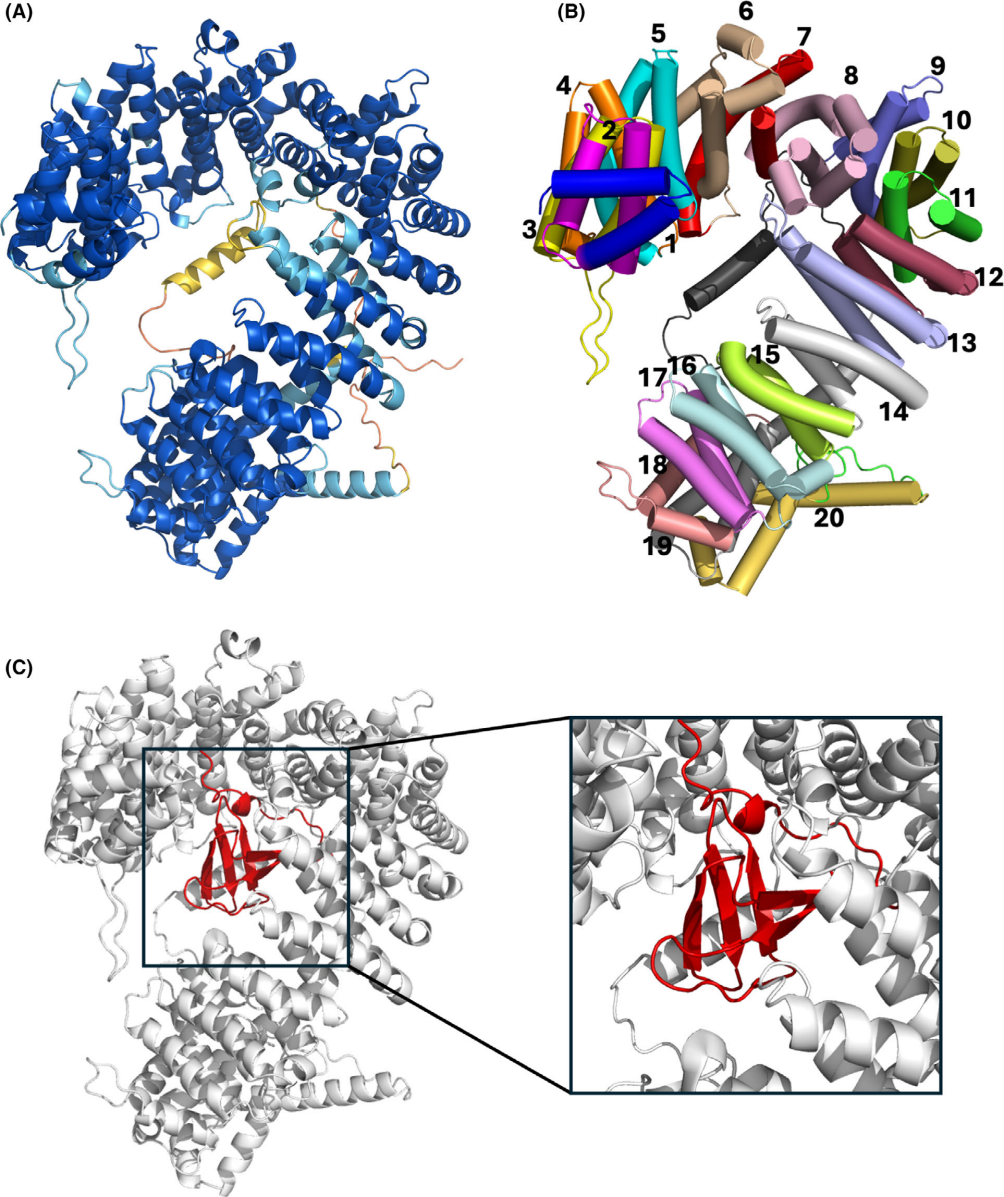
AlphaFold3 models of Imp7 and Imp7 in complex with IN CTD. (A) The model of Imp7 colored using AlphaFold model parameter. The overall model has high plDDT (plDDT>90, blue) with one region of low plDDT (70 > plDDT >50, yellow) and very few regions of very low plDDT (plDDT<50, orange). (B) Model of Imp7 is shown in tube representation to highlight its 20 HEAT repeats, which form the characteristic superhelical structure typical of β‐karyopherins (C) The model Imp7‐ IN CTD complex. IN CTD (red) in the core of Imp7 (gray), with an enlarged view of IN CTD in the binding site within the core of Imp7.

Structural models of Xenopus‐Imp7 and the complex of Xenopus‐Imp7 and IN C‐terminal domain (CTD) were generated using DeepMind's AlphaFold3, (https://alphafoldserver.com/) [44]. The IN CTD sequence was provided as a separate chain for complex modeling. The version 1.5.5 of AlphaFold was employed with default settings (Fig. 4). The predicted model of Imp7 displays the characteristic right‐handed superhelical architecture typical of karyopherin β family proteins. These proteins are composed of tandem HEAT repeats, each comprising two α‐helices connected by a flexible loop [45], and the Imp7 model conforms to this expected architecture (Fig. 4A). Specifically, the model contains 20 HEAT repeats that form a solenoid structure, curving inward to create a central cavity (Fig. 4B). The N‐terminal and central regions exhibit well‐packed HEAT repeat arrays, while the C‐terminal region appears less structured and more flexible, suggesting potential dynamic behavior in this part of the protein. The predicted TM‐score (pTM) of the model is 0.82, indicating an overall high confidence in the global fold. Furthermore, the majority of the predicted local distance difference test (pLDDT) scores exceed 90, suggesting high per‐residue confidence across most of the structure. However, a distinct region spanning residues 880–940 (61 amino acid residues) displays low pLDDT scores, indicating structural uncertainty and likely a disordered region. Such disorder may arise due to the fact that importins often bind their cargo and regulatory factors (e.g., RanGTP or other karyopherins) within the central core of their structure, and these interactions can induce substantial conformational rearrangements. The AlphaFold model of the Imp7–IN CTD complex positions IN CTD within the central cavity of Imp7, consistent with the known cargo‐binding mechanism of karyopherins (Fig. 4C). The overall architecture of Imp7 in the complex remains largely unchanged compared to the unbound Imp7 model, with a predicted TM‐score (pTM) of 0.77, indicating high structural similarity. Most residues in the complex model exhibit high confidence, with pLDDT scores above 90. Notably, the same region identified as disordered in the unbound Imp7 model (Residues 880–940) also remains undefined in the complex.

### The complex of Imp7 and IN exhibits high affinity

**Fig. 5:**
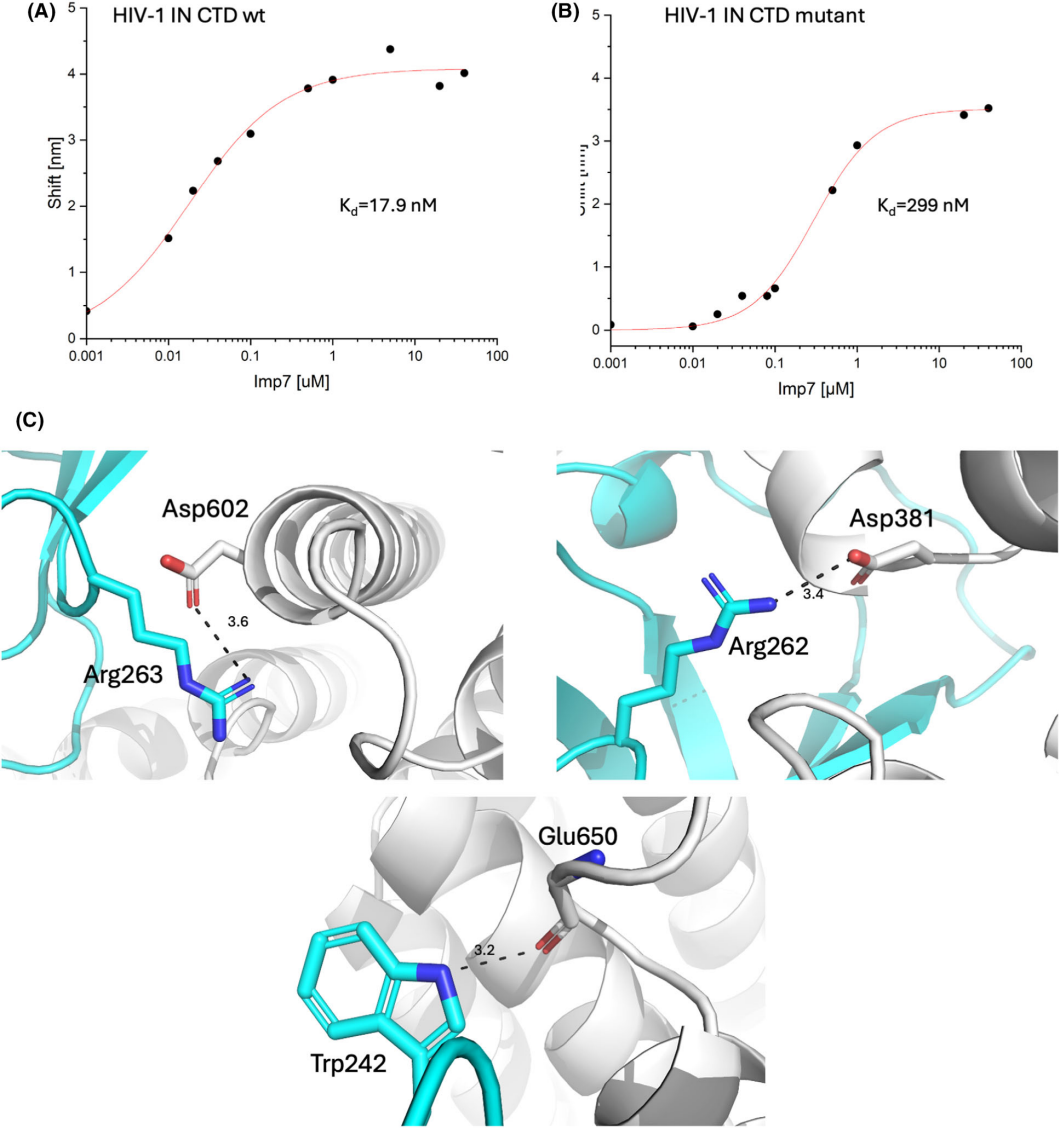
Affinity measurements of IN CTD wt and mutant for Imp7 using Bio‐Layer Interferometry and illustration of potential hydrogen bond between IN CTD and Imp7. (A) and (B) Steady‐state analysis of the binding interactions between Imp7 and (A) wt IN CTD and (B) mutant IN CTD. The wt IN CTD exhibits a K_d_ of 17.9 nm, indicating strong affinity for Imp7, as shown by the binding curve in panel (A) In contrast, the mutant integrase shows significant decreased binding affinity, with a K_d_ value of 299 nm, as shown in panel (B) The data represent steady‐state responses fitted to a 1:1 binding model. Binding curves representative of two independent experiments. (C) Examples of potential hydrogen bond interactions between the NLS of IN (shown in cyan) and Imp7 (shown in white), based on the AlphaFold3 model of the complex. The left Figure illustrates a possible hydrogen bond between Arg263 from the second NLS region of IN (a residue also targeted in the mutagenesis‐based affinity experiments) and Asp602 within the core of Imp7. The right figure highlights another predicted hydrogen bond interaction between residue Arg261 of IN and residue D381 of Imp7. The bottom panel shows a potential hydrogen bond between Trp242 from the first NLS region of IN and Glu650 main chain carbonyl of Imp7.

In addition to the structural analysis of the complex, we have conducted biochemical assays to quantitatively assess the strength of the interactions. We examined the binding affinity between the full length Imp7 and IN CTD (Residues 220–288). The interaction of the two components was assessed using biolayer interferometry. Imp7 and IN CTD showed high affinity, 1:1 stoichiometry with K_d_ value of 17.9 nm (Fig. 5A). This is particularly important for Imp7's role in transporting IN, as it facilitates the translocation of IN across the nuclear envelope. To examine the contribution of the NLS to the binding affinity, we have generated a mutant of the IN CTD in which the RRKAK motif was mutated to be RAAAK. This mutation significantly reduced the affinity of the interaction, yielding a K_d_ of 299 nm (Fig. 5B), compared to the WT construct clearly indicating that the NLS plays a critical role in mediating high‐affinity binding between IN and Imp7. Structural modeling further supports this conclusion. Examination of the AlphaFold generated model of the complex reveals that specific residues from the IN NLS are positioned to form potential hydrogen bonds with the Imp7 core (Fig. 5C).

Karyopherin–cargo interactions generally span a wide affinity range, from nanomolar to micromolar, depending on factors such as the specific karyopherin–cargo pair, the presence of cofactors such as RanGTP, and the nature of the cargo's nuclear localization or export signals. For instance, the interaction between Impβ and HIV‐1 Rev is characterized by a 2:1 stoichiometry and affinities ranging from 0.57 to 7.7 μm, reflecting the diversity and complexity of such transport systems [30]. Another example is Impα and transcription factor ChREBP (Carbohydrate‐Responsive Element‐Binding Protein), which contains an NLS recognized by Impα. Structural and biochemical studies have shown that the ChREBP NLS peptide binds Impα with a K_d_ of ~ 3 μm, primarily through hydrogen bonds and van der Waals interactions [46].

High‐affinity interactions in the low nanomolar range are also well‐documented, particularly for Impα–NLS cargo complexes. For example, the engineered NLS peptide Bimax2 binds Impα with a K_d_ of ~ 6 nm [47] and the interaction between Kap60p (the yeast homolog of Impα) and the NLS of the SV40 T‐antigen exhibits a K_d_ of 2 nm [48], highlighting the upper end of binding affinity within nuclear transport systems. In this context, the interaction between Imp7 and IN CTD falls within the low nanomolar range, placing it among the higher‐affinity karyopherin–cargo interactions. The high‐affinity binding observed between Imp7 and IN CTD further emphasizes the specificity and potential physiological significance of their interaction. Moreover, a strong interaction is essential to prevent dissociation during the transport. Once Imp7 delivers IN to the nucleus, the complex must dissociate, a process that requires energy and is mediated by RanGTP to overcome the strong binding [49, 50].

### Imp7 absence prevents IN translocation to nucleus in HEK cells

**Fig. 6:**
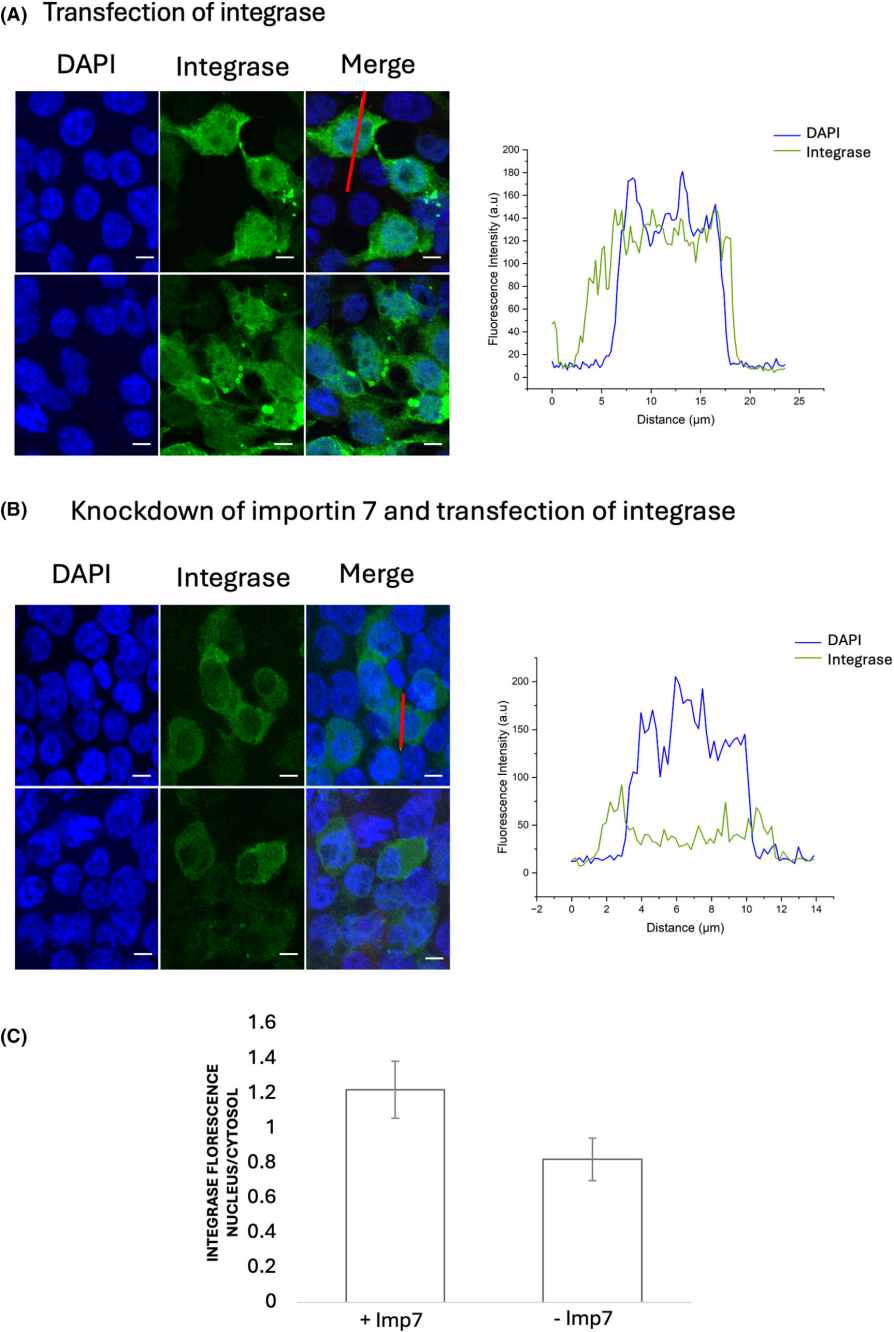
Expression and localization of full‐length IN in HEK293T cells. (A) Confocal microscopy of HEK293T cells transfected with IN. The cells were fixed, permeabilized, and immunostained with an anti‐HIV‐1 integrase antibody, followed by appropriate fluorescently labeled secondary antibodies to visualize the integrase. The cells were also stained with DAPI to mark DNA. Integrase is visible inside the nucleus. The red line represents the nucleus that used to generate a fluorescence intensity profile for integrase and DAPI for the graph on the right, demonstrating that integrase enters the nucleus very efficiently. (B) Confocal microscopy of HEK293T cells where endogenous Imp7 was knocked down and IN was simultaneously transfected. The cells were treated under identical conditions. The red line represents the nucleus that used to generate a fluorescence intensity profile for integrase and DAPI for the graph on the right, showing that when Imp7 is knocked down, the integrase does not enter the nucleus in the same manner, and its presence in the nucleus is significantly reduced. Images in A and B are representative of several images obtained. The scale bar in sections A and B indicates the length of 5 μm. (C) Ratio of nuclear‐to‐cytoplasmic fluorescence (F_n/c_). In cells expressing endogenous Imp7, F_n/c_ > 1, indicating that integrase is predominantly localized in the nucleus. In contrast, in Imp7‐knockdown cells, F_n/c_ < 1, demonstrating that integrase shows reduced nuclear entry and accumulated in the cytoplasm. The ratio was calculated for about 25 cells for each condition, and values represent the mean ± standard deviation. Statistical significance was determined using *t*‐test (*P* < 0.01).

To evaluate the role of Imp7 in the nuclear import of IN, immunofluorescence staining was performed in HEK cells expressing full‐length IN under two conditions: in the presence of endogenous Imp7 and following its knockdown. Under normal conditions, IN displayed strong nuclear localization, as shown by intense fluorescence concentrated within the nucleus (Fig. 6A), indicating efficient nuclear import. In contrast, when endogenous Imp7 was knocked down from the cells, the fluorescence signal was restricted mainly to the cytoplasm, with substantially reduced nuclear staining (Fig. 6B). Quantification of the fluorescence signal under normal conditions showed a nuclear‐to‐cytoplasmic fluorescence ratio (F_n/c_) greater than 1, consistent with predominant nuclear localization of IN, while under Imp7 knockdown condition, F_n/c_ was lower than 1, clearly indicating that IN is less capable of translocating into the nucleus when Imp7 levels are low (Fig. 6C).

### HDX‐MS analysis identifies that the critical regions of Imp7 interacting with IN are located within its core

**Fig. 7:**
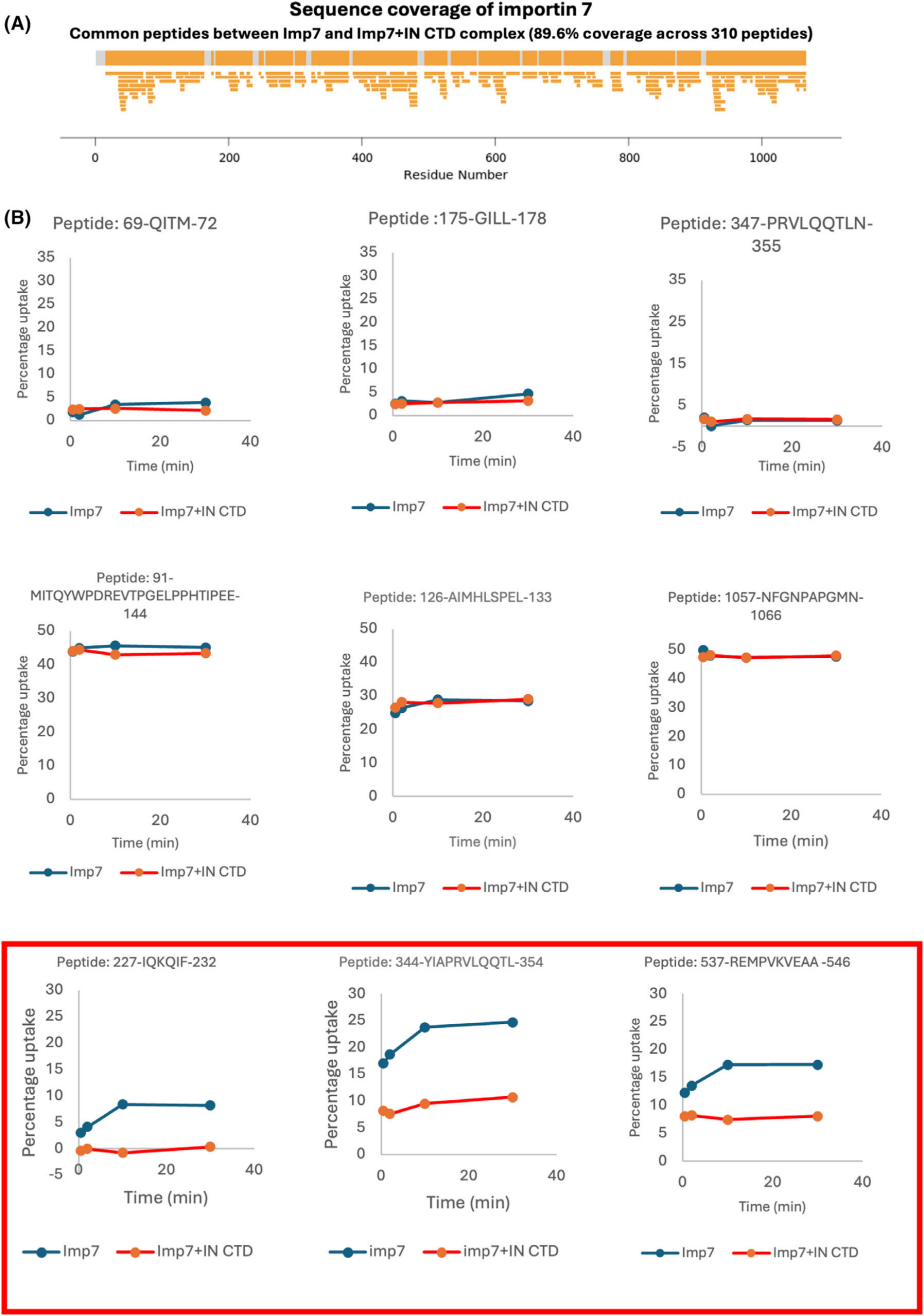
HDX‐MS analysis of Imp7 in unbound and IN bound states. (A) The sequence coverage of Imp7 achieved is 89.6%, based on 310 identified peptides. (B) The uptake plots show HDX data for different peptide states in Imp7, with blue representing the unbound state and red representing the complex state. The first row highlights peptides with no exchange in either state. The second row shows peptides with high exchange rates in both states. The third row, highlighted with a red rectangular, demonstrate peptides that exhibit different exchange rates between the unbound and bound states. In the bound state (red), the exchange is significantly reduced, indicating that these regions are interacting with IN CTD.

**Fig. 8:**
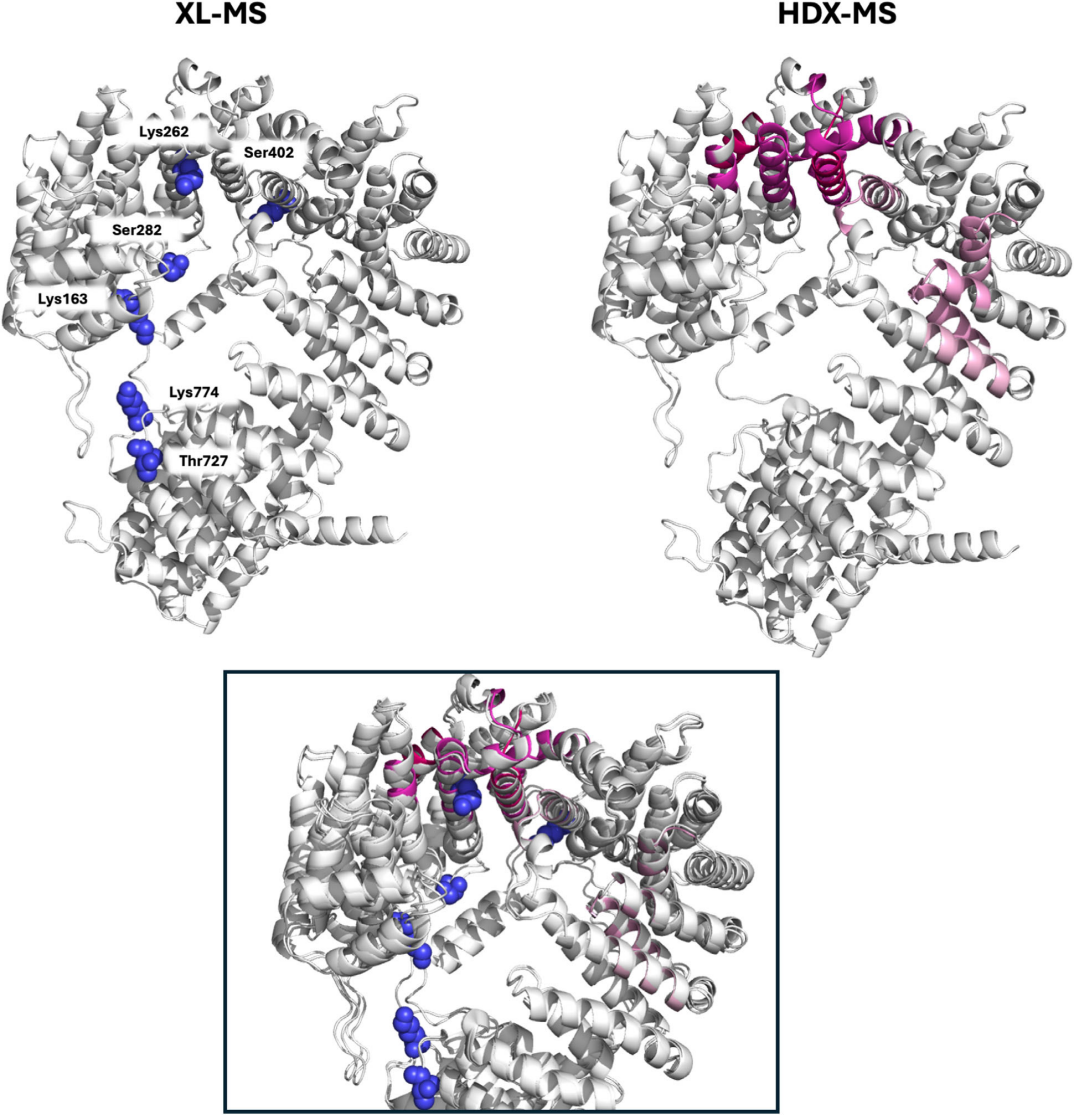
Mass spectrometry results of Imp7. The cross‐linking (left) and hydrogen‐deuterium exchange (right) are mapped onto the AlphaFold model of Imp7. Both XL‐MS and HDX‐MS data highlight the regions within the core domain of Imp7 that interact with IN CTD. The lower panel shows the XL‐MS and HDX‐MS results of Imp7 aligned on the AlphaFold model, showing a clear agreement. A few regions in the HDX are positioned very close to and even overlap with the cross‐linking sites.

To further characterize the interactions, HDX‐MS was performed for Xenopus‐Imp7 in the apo state and in complex with IN CTD. A total of 310 peptides were identified in the HDX‐MS results, mapping 89.6% of the Imp7 sequence (Fig. 7A). Peptides that are less than 10% deuterated can be regarded as practically buried in the structure and are not exposed to the solvent, hence do not H‐D exchange. For example, peptides 69–72, 175–178, and 347–355 have less than 10% deuteration both in the unbound and in the complex state; therefore, they are extremely buried in the structure of Imp7 in both apo and complex states (Fig. 7B). In contrast, peptides that are more than 10% deuterated can be regarded as exposed to the solvent. For instance, peptides located at either the N‐terminal or the C‐terminal regions of the protein exhibit a high deuteration percentage. This aligns well with the observation that these two regions are typically solvent exposed and flexible, and consequently undergo faster and more accessible exchange. In the context of the Imp7 and IN CTD interactions, the HDX‐MS results map larger regions in the Imp7 structure and provide more accurate information regarding the amino acids that are involved in this interaction. In this context, peptides 227–232, 273–296, 344–356, 355–379, 414–425, 537–546, 576–593, and 606–614 have significantly reduced HDX rates upon binding with IN CTD, compared to other peptides and are found in the core of Imp7 (Figs. 7 and 8). This suggests the involvement of these regions in interactions.

### XL‐MS analysis supports the role of the NLS in IN

**Fig. 9:**
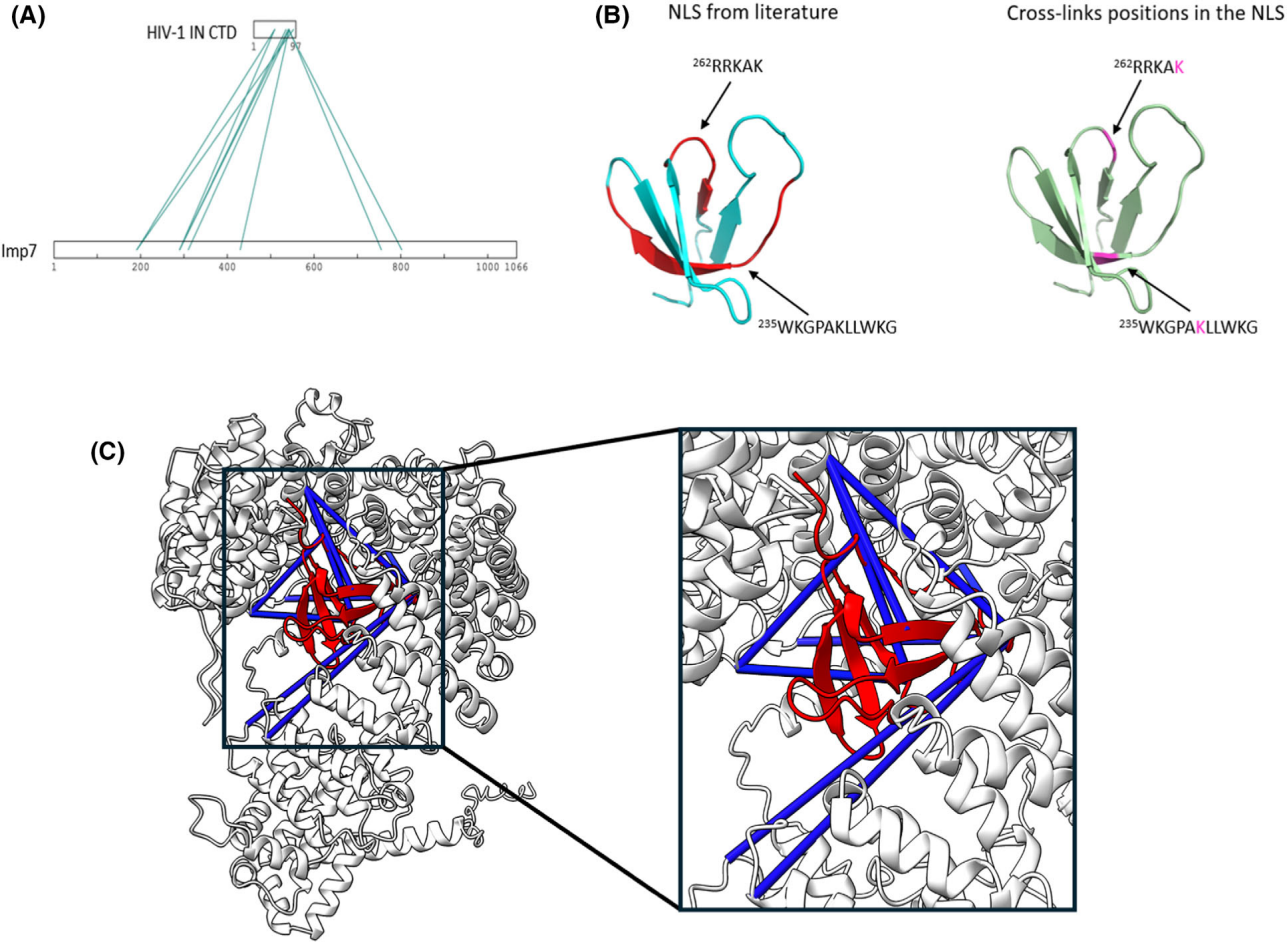
XL‐MS results. (A) Two‐dimensional map of the crosslinks between IN CTD and Imp7. Within the IN CTD, the cross‐links predominantly occur in two distinct regions, both situated within the nuclear localization signal (NLS) of the protein. (B) Three‐dimensional structure of IN CTD (PDB ID: 1EX4), with the NLS highlighted in red on the left, and two out of the five cross‐linking regions indicated in pink on the right. (C) Representation of the XL‐MS results (blue lines) on the AlphaFold model of the complex (IN CTD in red and Imp7 in white). The model incorporating the cross‐links was generated using the xlms‐tools [74].

XL‐MS analysis was also employed in order to cross‐validate the results obtained from the structural predictions as well as the HDX‐MS analysis. The complex of Xenopus‐Imp7 and IN CTD was crosslinked using BS3 (bis(sulfosuccinimidyl)suberate), an amine‐amine crosslinker, targeting mainly primary amines of lysines and N termini. In addition to lysine residues, BS3 can react with hydroxyl groups of serine, threonine, and tyrosine side chains; however, with lower efficiency [51]. Identification of crosslinks was conducted with Merox 2.0 [52], followed by manual examination of their MS/MS spectra to ensure stringent quality control. Only crosslinks with good fragmentation (identifying more than half of both peptides) and containing at least one fragment with the crosslinker mass were retained. The identified crosslinks were mapped on the protein sequence and visualized with the xiNET (Fig. 9A) [53]. We identified 31 high‐quality crosslinks at 1% false discovery rate (FDR), across Imp7 and IN CTD. The majority of the crosslinks (20) are intra‐molecular and some are bridging Imp7 and IN CTD. A total of 11 inter‐crosslinks were identified, including 2 duplicates, resulting in 9 unique crosslinks within the protein complex (Table 1). Notably, this analysis revealed that IN CTD interacts with multiple regions across Imp7, supporting its high affinity binding. Specifically, out of the 9 unique cross‐links identified, two are located within the NLS of IN, at Lys240 and Lys246 (Fig. 9B). These results are consistent with the previous observation that the NLS is important for Imp7 binding, and in line with its known role in mediating nuclear import and supporting the notion that Imp7 directly recognizes the NLS to facilitate nuclear transport. Additionally, XL‐MS analysis showed that other regions (between residues 200–400, and in the vicinity of residue 800), primarily within the core of Imp7, are involved in the interaction with IN CTD, in agreement with the AlphaFold3 model (Fig. 4B and 9C). Comparison between XL‐MS and HDX‐MS results shows overall agreement in identifying the Imp7 core as the interaction region with IN CTD (Fig. 8). Although the exact sequences highlighted by each method do not fully overlap, this difference is expected given the distinct principles underlying the two approaches. HDX‐MS reports on changes in solvent accessibility and conformational dynamics, whereas XL‐MS captures proximity between specific residues also depending on the cross‐linker. Despite these methodological differences, both datasets consistently indicate that the majority of contacts with IN CTD are in the core region of Imp7 (Fig. 8). Additionally, we observed cross‐links involving a region of Imp7 (Lys774 and Thr727) that, according to the AlphaFold3 model, is located distal to both the core and IN CTD. We thus assume that upon IN binding, Imp7 undergoes a conformational change accommodating itself to the cargo by bringing this region into proximity with its cargo. This is consistent with the known structural plasticity of importins and their ability to adapt diverse conformations for cargo recognition [9, 11, 54, 55, 56].

**Table 1:** List of the cross‐links between Imp7 and IN CTD (residue numbering is based on the unmodified native sequence).

Residue and position in Imp7	Residue and position in IN
Lys163	Lys240
Lys262	Lys240
Lys163	Ser283
Thr727	Lys273
Lys262	Lys266
Lys262	Tyr271
Ser282	Lys273
Ser402	Lys273
Lys774	Tyr271

## Discussion

The study of the interaction between Importin 7 (Imp7) and HIV‐1 integrase (IN) is aimed to better understand the initial stage of IN viral nuclear import. Beyond their established role in transporting essential cellular proteins, karyopherins such as Imp7 are also exploited by viral proteins to facilitate entry into the nucleus. A prominent example is the direct interaction between Imp7 and IN, which enables translocation through the nuclear pore complex. Given the importance of this process for successful viral infection and subsequent replication, revealing the structural details of the Imp7–IN complex has therapeutic potential. Our study provides new insights into this interaction and further underscores the critical role of Imp7 in IN nuclear import.

The calculated model, supported by our experimental results of HDX‐MS and XL‐MS, positions IN CTD in the central cavity of Imp7 (Fig. 4C and 8). This is consistent with the typical mode of cargo binding also observed in other karyopherins. In this regard, in the Impβ–RanGTP complex, RanGTP is typically located within the cavity of the importin, stabilized by extensive electrostatic and hydrophobic interactions [56]. Similarly, structures of exportin–cargo–RanGTP complexes show the cargo positioned deeply in the karyopherin cavity and its strong interactions driven by shape complementarity and flexible domain adaptation [57, 58]. For this reason, our results are supported by a conserved mode of binding within the karyopherin family, where the superhelical architecture provides a binding site for cargo binding. In addition, karyopherins can either bind their cargo directly forming a 1:1 complex or function as a transport complex involving two different importins that bind the cargo. For example, Impα and Impβ can form a transport complex with some cargos or act independently by binding their corresponding cargo directly [11, 59]. Impβ can also act as an adapter for Imp7 when binding H1 histone [18, 19, 60]. In this case, Impβ serves as the direct transporter, interacting with H1 histone and functioning as an adaptor between H1 histone and Imp7. In contrast, our results demonstrate that, in the case of IN, Imp7 directly binds to and transports IN into the nucleus.

In addition to structural and biochemical analyses of the Imp7‐IN CTD interaction, we have shown that the presence of imp7 is essential for nuclear import of IN. When HEK cells express their endogenous Imp7, IN is transported into the nucleus, demonstrating that even in the absence of the viral capsid, IN is imported into the nucleus. Knockdown of endogenous Imp7 from HEK cells significantly reduced the nuclear localization of IN in the nucleus (Fig. 6), underscoring again the specificity and necessity of Imp7 in the IN nuclear import. These outcomes suggest that targeting the Imp7‐IN interaction *in‐vivo* could serve as a viable strategy for inhibiting HIV‐1 replication by preventing the viral integration step, offering new avenues for antiviral therapy development.

In 2024 1.3 million people were infected with HIV, contributing to a global total of over 40 million individuals being HIV‐positive [61]. Although advances in treatments have transformed AIDS into a manageable chronic condition, HIV remains a lifelong infection that could be transmitted with significant impacts on overall health [62, 63]. Consequently, there is an ongoing need to develop new approaches to address the permanence of HIV infection. Although recent studies indicate that capsid uncoating occurs in the nucleus rather than the cytoplasm, our findings highlight that viral protein–host factor interactions remain critical when uncoating takes place in the cytoplasm or when partially uncoated viral complexes are present, providing a complementary mechanism that supports efficient infection. Understanding the role of Imp7 in the nuclear transport of IN could open new avenues for therapeutic intervention. Since IN is essential for integrating the viral genome into the host DNA, a critical step in the HIV replication cycle, disrupting its nuclear import represents a promising strategy for halting disease progression. Targeting the Imp7‐IN interaction and disturbing it by using small molecules that bind to IN and occupy its interface with Imp7 could prevent the establishment of a permanent viral reservoir, complementing current antiretroviral therapies that act after integration has occurred [64, 65]. The concept of targeting intracellular protein–protein interactions with small molecules is an established and clinically validated strategy. Several FDA‐approved drugs exemplify the therapeutic potential of this approach. For example, venetoclax, a small, lipophilic molecule designed to passively diffuse across the cell membrane, targets the protein BCL‐2, a regulatory protein that plays a key role in controlling apoptosis, by mimicking the BH3 domain of pro‐apoptotic proteins, thereby competitively inhibiting BCL‐2's interactions with its binding partner [66, 67, 68]. To be effective, venetoclax must enter the cytosol, where it binds BCL‐2 and promotes apoptosis. Another example is the Nutlin‐3 derivatives that disturb the interactions between MDM2 and p53, which can trigger apoptosis [69, 70]. These examples demonstrate that rationally designed small molecules can effectively penetrate cells and modulate intracellular protein–protein interactions and act as a therapeutic strategy. This strategy could also be applied to disrupt the Imp7–IN interaction. Further research into the specific molecular interactions between Imp7 and IN will be vital for the development of such approaches, potentially paving the way toward a functional cure.

Currently, several integrase inhibitors, used in combination therapies such as Stribild and Triumeq, effectively suppress HIV replication by targeting IN catalytic core domain [71, 72, 73]. However, these treatments do not eliminate the virus from the body. Targeting the interaction between IN and Imp7 offers an alternative therapeutic approach by preventing nuclear import of the viral pre‐integration complex. Disrupting this interaction through small peptides could trap the complex in the cytoplasm, blocking infection and aiding in viral clearance. Given the specificity and essential nature of the Imp7–IN interaction, it represents a promising target for intervention. Our study, combined with further analysis of this interaction at the molecular level as well as the identification of inhibitors disrupting the complex, could become a viable approach in the toolkit of anti‐HIV therapy.

## Conclusions

In conclusion, we have shown that Imp7 binds IN tightly and has a role in its nuclear transport. This study adds another layer to the complex mechanism of HIV‐1 infection. While capsid‐mediated nuclear transport is now recognized as the primary pathway for delivering HIV‐1 components into the nucleus, the strong interaction between Imp7 and HIV‐1 IN provides a complementary route. This alternative route may become particularly relevant when the viral capsid disassembles prematurely in the cytoplasm rather than at the nuclear pore. In such circumstances, Imp7‐mediated transport of IN could help ensure efficient nuclear entry of viral components, thereby promoting successful establishment of infection.

## Conflict of interest

The authors declare no conflict of interest.

## Author contributions

OL and JB conceived and designed the project. JB, AY, TO, HA, OL, and DR acquired and analyzed the data. JB, and OL interpreted the data. JB, OL, TO, and DR wrote the paper.

## Data Availability

The crosslinking (XL‐MS) and hydrogen–deuterium exchange (HDX‐MS) datasets have been deposited in the PRIDE repository and are publicly accessible upon publication. The XL‐MS accession number is PXD066885 and the HDX‐MS accession number is PXD068263. The AlphaFold models described in this study can be generated using the AlphaFold Server. Additional data supporting the findings of this work are available from the corresponding author upon request.
